# Wnt/β-catenin signalling: function, biological mechanisms, and therapeutic opportunities

**DOI:** 10.1038/s41392-021-00762-6

**Published:** 2022-01-03

**Authors:** Jiaqi Liu, Qing Xiao, Jiani Xiao, Chenxi Niu, Yuanyuan Li, Xiaojun Zhang, Zhengwei Zhou, Guang Shu, Gang Yin

**Affiliations:** 1 Department of Pathology, Xiangya Hospital, School of Basic Medical Sciences, Central South University, Changsha, 410013 China; 2 Department of Histology and Embryology, School of Basic Medical Sciences, Central South University, Changsha, 410013 China; 3 China-Africa Research Center of Infectious Diseases, School of Basic Medical sciences, Central South University, Changsha, 410013 China

**Keywords:** Oncogenes, Drug regulation, Cancer genetics, Non-coding RNAs, Self-renewal

## Abstract

The Wnt/β-catenin pathway comprises a family of proteins that play critical roles in embryonic development and adult tissue homeostasis. The deregulation of Wnt/β-catenin signalling often leads to various serious diseases, including cancer and non-cancer diseases. Although many articles have reviewed Wnt/β-catenin from various aspects, a systematic review encompassing the origin, composition, function, and clinical trials of the Wnt/β-catenin signalling pathway in tumour and diseases is lacking. In this article, we comprehensively review the Wnt/β-catenin pathway from the above five aspects in combination with the latest research. Finally, we propose challenges and opportunities for the development of small-molecular compounds targeting the Wnt signalling pathway in disease treatment.

## Introduction

Since the first member of the Wnt family was identified in 1982,^1^ studies on Wnt signalling have been steadily increasing. Notably, the Wnt/β-catenin signalling pathway is necessary for embryonic development and adult tissue homeostasis regeneration. Abnormal regulation of the pathway is closely associated with different diseases, suggesting that the Wnt/β-catenin signalling pathway is an attractive target for disease treatment. First, this article reviews non-tumour diseases and conditions related to the Wnt/β-catenin signalling pathway, such as hair loss, pigment disorders, wound healing, bone diseases, neurodegenerative diseases, and chronic obstructive pulmonary diseases. Second, we comprehensively summarize tumour diseases based on their biological features, including cancer stem cells, cancer metastasis, cancer metabolism, and cancer immunity. Finally, we review the role of Wnt/β-catenin in the diagnosis and clinical treatment of cancer and non-cancer diseases.

## Introduction to Wnt/β-catenin signalling

### Overview of Wnt/β-catenin signalling

The Wnt gene was originally derived from integrase-1 in mouse breast cancer and the wingless gene of *Drosophila*. Because the two genes and functional proteins are similar, researchers combined the terms as the Wnt gene.^1^ The Wnt signalling pathways include noncanonical and canonical pathways. The noncanonical Wnt pathways are independent of β-catenin-T-cell factor/lymphoid enhancer-binding factor (TCF/LEF), such as the Wnt/Ca^2+^ pathway and noncanonical Wnt planar cell polarity.^2^ The canonical Wnt pathway, also known as the Wnt/β-catenin pathway, involves the nuclear translocation of β-catenin and activation of target genes via TCF/LEF transcription factors. The canonical Wnt pathway mainly controls cell proliferation, whereas the noncanonical Wnt pathways regulate cell polarity and migration, and the two main pathways form a network of mutual regulation. Wnt signalling plays an important role in the self-renewal of some tissues in mammals. For example, the Wnt signalling pathway is related to the development and renewal of small-intestinal epithelial tissue and promotes the differentiation of Paneth cells at the base of the crypt. In addition, the Wnt signalling pathway is closely related to liver metabolism and regeneration, lung tissue repair and metabolism, hair follicle renewal, haematopoietic system development, and osteoblast maturation and activity.^3–5^

The Wnt/β-catenin pathway comprises four segments: the extracellular signal, membrane segment, cytoplasmic segment, and nuclear segment. Extracellular signals are mainly mediated by Wnt proteins, including Wnt3a, Wnt1, and Wnt5a. The cell membrane segment mainly contains the Wnt receptors Frizzled (specific sevenfold transmembrane receptor Frizzled protein) and LRP5/6. The cytoplasmic segment mainly includes β-catenin, DVL, glycogen synthase kinase-3β (GSK-3β), AXIN, APC, and casein kinase I (CK1). The nuclear segment mainly includes β-catenin, which translocates to the nucleus, TCF/LEF family members, and β-catenin downstream target genes, such as MMPs and c-Myc.^6^

**Fig. 1:**
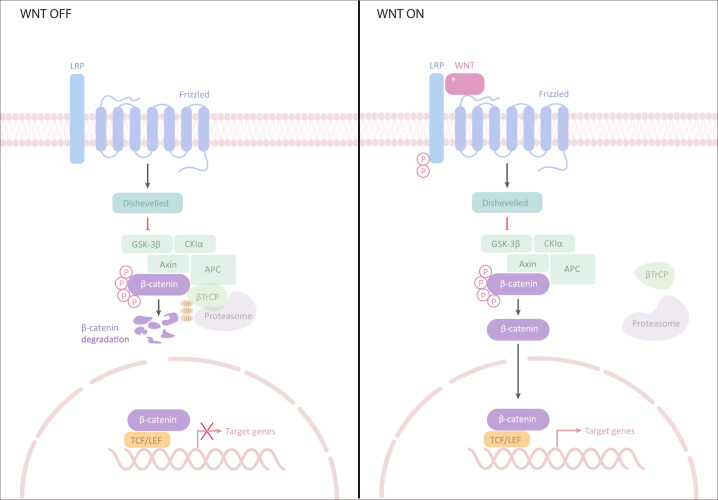
Wnt/β-catenin pathway. Left (inactivation of Wnt signalling): In the absence of Wnt signalling, β-catenin is degraded by protein complexes, including AXIN, APC, serine/threonine kinase GSK-3, and CK1 and E3 ubiquitin ligase β-trcp. Right (activation of Wnt signalling): Wnt signalling is activated by binding to its receptor, which induces the binding of AXIN to phosphorylated lipoprotein receptor-related protein (LRP). The destruction complex is broken, and then β-catenin stabilizes and binds to TCF in the nucleus to regulate the target gene. GSK-3 glycogen synthase kinase-3, AXIN axis inhibition protein, CK1 casein kinase 1, APC adenomatous polyposis coli, TCF T cell factor, LEF lymphocyte enhancer factor-1

The canonical Wnt pathway is usually highly conserved and activated via the binding of extracellular Wnt ligands to membrane receptors by autocrine/paracrine methods. Once activated, the typical Wnt pathway induces the stability of β-catenin and transfers it to the nucleus, ultimately facilitating the expression of genes involved in cell proliferation, survival, differentiation, and migration (Fig. 1).^7^

In the absence of Wnts, the transmembrane receptors FZD and LRP5/6 are located on the plasma membrane separately. In the cytoplasm, a “destruction complex” comprising adenomatous polyposis coli (APC), AXIN, casein kinase 1 (CK1) and glycogen synthase kinase 3 protein (GSK3 protein) captures β-catenin by phosphorylating CK1 and GSK3, thus activating the process of β-catenin degradation. Therefore, GROUCHOU, which binds to TCF/LEF, inhibits the transcription of target genes.

When Wnts are recognized by FZD and LRP5/6, the “destruction complex” is recruited to the cell membrane by interacting with FZD, which loses the ability to degrade β-catenin. The induced β-catenin translocates to the nucleus and activates the transcription of target genes by interacting with TCF/LEF. The cytoplasmic-nuclear shuttling of β-catenin is considered an important feature of Wnt/β-catenin pathway activation.^8,9^

Here, we review the diseases caused by abnormal Wnt/β-catenin signalling and the underlying mechanism, and we discuss the function of the components and regulators of the Wnt/β-catenin signalling pathway.

### Components and regulators of the Wnt/β-catenin pathway

**Fig. 2:**
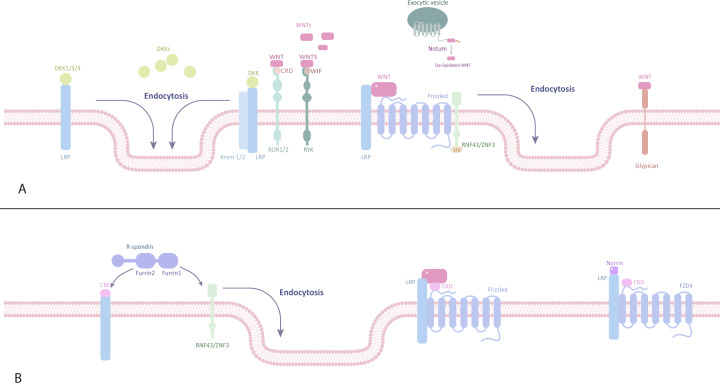
Inhibitors and activators of the Wnt/β-catenin pathway. **A** Inhibitors of the Wnt/β-catenin signalling pathway. sFRPs, WIF, Tiki, and Notum are located outside the plasma membrane and inhibit Wnt signal transduction by interacting with Wnts. FZD, LRP5/6, RNF43/ZNRF3, Krem-1/2, ROR, RYK, and glypican are located on the plasma membrane. RNF43/ZNRF3 binds to the FZD receptor complex and induces its ubiquitination and endocytosis. ROR, RYK, and glypican bind to Wnts and inhibit Wnt signal transduction. DKK1/3/4 binds to LRP5/6 or further forms a complex with Krem-1/2 to induce endocytosis. Groucho is located in the nucleus and inhibits the transcription of target genes by binding to LEF. **B** Activators of the Wnt/β-catenin signalling pathway. R-spondin and Norrin are located outside the plasma membrane. FZD, LRP5/6, RNF43/ZNRF3, and LGR4/5 are located on the plasma membrane. The β-catenin destruction complex (comprising axin, APC, GSK-3β, CK-1α, and β-catenin) and PP2A are located in the cytoplasm. TCF/LEF is located in the nucleus. Association of LGR4/5 and RNF43/ZNRF3 with R-spondin induces membrane retention of FZD. Norrin activates signal transduction by acting as a mimic of Wnts. PP2A dephosphorylates β-catenin to promote the accumulation of β-catenin in the cytoplasm. Β-catenin enters the nucleus and combines with TCF/LEF to initiate the transcription of target genes. DKK Dickkopf, WIF Wnt inhibitory factor, CRD cysteine-rich domain, LRP lipoprotein receptor-related protein, RNF ring finger protein

In this section, we first outline the components (Fig. 2) and functions of Wnt/β-catenin signalling (Table 1), followed by other molecules and pathways affecting the Wnt/β-catenin pathway (Table 2). Finally, we show that several newly discovered components interact with the Wnt/β-catenin pathway.

**Table 1:** Main components of the Wnt/β-catenin signalling pathway

Components	Role in WNT/β-catenin pathway	
Wnt	Binding with FZD/LRP receptor complex and activates Wnt/β-catenin siginal pathway.	
PORCN	An enzyme adds the palmitoleate group to Wnt proteins.	
WLS	A chaperone protein promotes the migration of Wnt proteins to the plasma membrane.	
FZDs	Forming a receptor complex together.	Bind to Wnt proteins and activates Wnt/β-catenin siginal pathway.
LRP5/6		
Dvl	An essential component in the transduction of Wnt signalling to the intracellular compartment.	
AXIN	Forming the β-catenin destruction complex together.	Scaffold protein.
APC		
CK-1α		Phosphorylation of β-catenin (Ser45).
GSK-3β		Phosphorylation of β-catenin (Thr41, Ser33, Ser37).
β-catenin		A central molecule in the WNT/β-catenin signalling pathway.
TRCP-1	Binding with phosphorylated β-catenin and degrades it. (Off-state)	
Groucho	Transcriptional repressor which can be bound with the TCF and silences the TCF-mediated gene expression. (Off-state)	
TCF/LEF	Transcriptional activating factor which can be bound with β-catenin and activate target gene transcription. (On-state)	
WIF	Binding with Wnt proteins and prevent its interaction with the receptor complex.	
sFRPs		
Glypican-3	Binding Wnt proteins to the cell surface, regulating their extracellular distribution and ssignalling activity.	
Tiki	Processing some of the Wnt proteins thereby rendering them inactive.	
Notum		
R-spondin	The ligands for LGR4/5 and upregulation of Wnt signalling.	
Norrin	The ligands for LGR4/5 and upregulation of Wnt signalling.	
LGRs	The binding of LGR5/6 and R-spondin initiates the formation of a complex with RNF43 or ZNRF3.	
RNF43	E3 ligases which can induce the endocytosis of the FZD proteins and downregulate Wnt signalling.	
ZNRF3		
DKKs	DKK1/2/4 can bind to LRP5/6 and downregulation of Wnt signalling.	
ROR	Receptor of Wnt proteins.	
RYK		
PP2A	Dephosphorylating AXIN and β-catenin and thereby increasing the number of β-catenin.	
PP1		

The protein information is from https://www.UniProt.org/

**Table 2:** Factors to upregulate or downregulate Wnt/β-catenin signalling

Molecules to regulate the activation of Wnt/ /β-catenin signalling				
Molecules	Target	Inhibitor/activator of the target	Effect on Wnt signalling	Reference
R-spondin	ZNRF3/RNF43	Inhibitor	Activates	^29,37^
syndecan-1	Wnts, R-spondin	Activator	Activates	^321^
Norrin	FZD4, LRP5/6	Activator	Activates	^38,39^
ADNP	β-catenin	Activator	Activates	^322^
Notum	Wnt	Inhibitor	Inhibits	^323^
Tiki	Wnt	Inhibitor	Inhibits	^323^
DKKs	LRP5/6	Inhibitor	Inhibits	^108^^,324^
Wise/SOST	LRP5/6	Inhibitor	Inhibits	^324^
IGFBP-4	FZD8, LRP6	Inhibitor	Inhibits	^325^
sFRPs	Wnt	Inhibitor	Inhibits	^326^
WIF1	Wnt	Inhibitor	Inhibits	^327^
ZNRF3/RNF43	FZD	Inhibitor	Inhibits	^328^

MicroRNA to activate or inhibit wnt signalling					
MicroRNA	Target	Inhibitor/activator of the target	Effect on Wnt signalling	Disease	Reference
miR-100/miR-125b	DKK1/3, ZNRF3/RNF43, APC2	Inhibitor	Activates	Colorectal cancer	^43^
miR-31	DKK1, GSK3β, AXIN1	Inhibitor	Activates	1. breast tumorigenesis 2. inflammatory bowel diseases	^44,45^
miR-455-3p	DKK3, GSK3β	Inhibitor	Activates	Esophageal squamous cell carcinoma	^46^
miR-29	GSK3β, CTNNBIP1, HBP1, GLIS2	Inhibitor	Activates	Pathologic hypertrophy and fibrosis of the myocardium	^47^
miR-1246	AXIN2, GSK3β	Inhibitor	Activates	Hepatocellular carcinoma	^48^
miR-128-3p	AXIN1, SFRP2, WIF1	Inhibitor	Activates	Non-small cell lung cancer (NSCLC)	^49^
miR-374a	WIF1, PTEN, WNT5A	Inhibitor	Activates	Breast cancer metastasis	^50^
miR-22-3p/5p	SFRP2, PCDH15	Inhibitor	Activates	Glioblastoma	^51^
miR-106a-3p	APC	Inhibitor	Activates	Gastric cancer	^52^
miR-494	APC	Inhibitor	Activates	Colorectal cancer	^53^
miR-155	APC	Inhibitor	Activates	Hepatocellular carcinoma	^54^
miR-92a-3p	FBXW7, MOAP1	Inhibitor	Activates	Colorectal cancer	^55^
miR-182-5p	FOXO3a	Inhibitor	Activates	Hepatocellular carcinoma	^56^
miR-199a-5p	FZD4, JAG1, WNT2	Inhibitor	Inhibits	Duchenne muscular dystrophy (DMD)	^57^
miR-384-5p	FZD1, FZD2, Tgrbr1, Lrp6	Inhibitor	Inhibits	Inhibits cardiac fibrosis	^58^
miR-4496	β-catenin	Inhibitor	Inhibits	Inhibits intestinal tumorigenesis	^59^
miR-181a-5p	β-catenin, TCF4	Inhibitor	Inhibits	Inhibits colorectal cancer	^60^
miR-125b-5p	STAT3	Inhibitor	Inhibits	Inhibits hepatocellular carcinoma	^61^
miR-200c	FUT4	Inhibitor	Inhibits	Infertility and abortion.	^62^
miR-708-5p	CDH1	Activator	Inhibits	Inhibits lung cancer stem cell-like phenotypes	^63^

LncRNA to activate or inhibit wnt signalling					
LncRNA	Target	Inhibitor/activator of the target	Effect on Wnt signalling	Disease	Reference
FAST	β-TrCP	Inhibitor	Activates	/	^64^
JPX	GSK-3β	Inhibitor	Activates	Lung cancer	^65^
LALR1	AXIN1	Inhibitor	Activates	/	^66^
Linc00210	CTNNBIP1	Inhibitor	Activates	Liver cancer	^67^
AP-2α	CCAL	Inhibitor	Activates	Colorectal cancer	^68^
NEAT1	DDX5	Activator	Activates	Colorectal cancer	^69^
Lnc-β-Catm	β-catenin	Activator	Activates	Liver cancer	^70^
MIR100HG	miR-100/miR-125b	Activator	Activates	Colorectal cancer	^43^
MIR22HG	miR-22-3p/5p	Activator	Activates	Glioblastoma	^51^
CRNDE	miR-181a-5p	Inhibitor	Activates	Colorectal cancer	^60^
LINC01133	miR-106a-3p	Inhibitor	Inhibits	Inhibits gastric cancer	^52^

Crosstalk between Wnt/β-catenin and other pathways				
Pathway	Effector	Target	Effect on Wnt signalling	Reference
BMP	BMP2	SOST, DKK1	Inhibits	^329,330^
PI3K/AKt/mTOR	mTORC1	DVL	Inhibits	^331^
Hippo	TAZ	DVL	Inhibits	^332^
Hedgehog	/	β-catenin	Inhibits	^333^
p53	RARRES3, p53	Cyclin D, AXIN2, c-Myc, Wnt1/6/7a	Inhibits	^334,335^
TGF-β	TGF-β	Wnts, DKK1	Activates	^113^^,336^
Ras/Raf/Mek/Erk	Ras	β-catenin	Activates	^337–339^
Notch	Notch1/Notch2	PROX1, AXIN2, c-Myc, APCDD1, β-catenin/GSK3-β, C1q	Inhibits/activates	^340–343^

#### Components of the Wnt/β-catenin signalling pathway and their functions

Wnts are secreted proteins, and 19 Wnt proteins have been identified in the human body.^10^ Among them, Wnt3a and Wnt1 act as ligands to activate the Wnt/β-catenin signalling pathway after translation and modification. Posttranslational modification of Wnts mainly includes lipid modification and glycosylation, and lipid modification is performed by PORCN. PORCN, located in the endoplasmic reticulum, can add palmitoleate groups to Wnt proteins, and it is a member of the membrane-bound O-acyltransferases (MBOATs). Lipid modification is necessary for Wnt activity, and the opposite is true for glycosylation.^11^ WLS encodes a multipass transmembrane protein that localizes to the Golgi, is recycled between the Golgi and plasma membrane^12^ and transports lipid-modified Wnts from the endoplasmic reticulum to the cell surface.

The other main components of the Wnt/β-catenin signalling pathway include FZD, LRP5/6, DvL, AXIN, APC, GSK-3β, CK-1α, β-catenin, and TCF/LEF. FZD proteins are a family of seven transmembrane (TM) receptors and are located on the plasma membrane.^13^ The cysteine-rich domain (CRD) is an extracellular domain at the N-terminal side of FZD proteins and is considered the site of interaction with Wnt ligands. The fully conserved KTxxxW domain is an intracellular domain at the C-terminal side of FZD proteins and interacts with the protein-95/discs-large/zona occludens-1 (PDZ) domains of DvL proteins.^14^ LRP5/6 are coreceptors of Wnts and are located on the plasma membrane. The intracellular part of LRP5/6 contains multiple phosphorylation sites, the phosphorylation of which is a key step in the initiation of signal transduction via Wnt/β-catenin signalling.^15^ DVL proteins located in the cytoplasm are pivotal in the transduction of WNT signalling from the receptor level to the intracellular compartment and contain three highly conserved domains: the DIX domain at the N-terminal side (the site interacts with AXIN), the PZD domain in the central part, and the DEP domain at the C-terminal side.^16^ When the Wnt ligand binds to the receptor, most of the DVL is recruited to the plasma membrane. Activated DvL guides the clustering of FZD and LRP6 and promotes the phosphorylation of LRP6.^17–19^ In addition, activated DVL recruits AXIN and GSK-3β to the plasma membrane and inhibits their functions.^18,20^ The DEP domain not only binds to FZD but can also change its conformation and thereby dimerize, triggering the WNT/FZD signalosome.^14^

When the Wnt/β-catenin signalling pathway is in the off-state, a β-catenin destruction complex (DC) is formed in the cytoplasm, comprising AXIN, the APC protein, GSK-3β, CK-1α, and β-catenin. AXIN functions as a scaffold protein because it contains binding domains for other components of the DC, such as the DIX domain at the C-terminal side and RGS domain at the N-terminal side (the site interacts with the APC protein).^14^ The APC protein is located in the cytoplasm, and mutations in this protein cause Wnt pathway activation in human cancers. Current models of APC action emphasize its role as a component of DCs to promote β-catenin degradation. Previous studies have shown that APC2 can only compensate for the role of APC as a component of DCs in promoting β-catenin degradation.^21^ Saito-Diaz et al. found that APCs transduce Wnt signals through clathrin-mediated endocytosis via the clathrin endocytic pathway without the Wnt ligand in APC-depleted cells. Concurrently, they proposed that APC inhibited the formation of constitutive, ligand-independent, clathrin-dependent signalosomes and the unnecessary activation of the Wnt pathway.^22^ Wnt ligands induce the phosphorylation of LRP6 receptors, mainly mediated by GSK-3β and CK-1α, and dual phosphorylation promotes the binding of LRP6 to AXIN. GSK-3β and CK-1α also induce the phosphorylation of β-catenin in the absence of Wnt ligands. Phosphorylated β-catenin is released from DCs and bound by Trcp-1 (also known as β-Trap) for ubiquitination and subsequent degradation.^23^

#### Other molecules and pathways affect the Wnt/β-catenin pathway

The canonical Wnt signalling pathway is regulated by many factors. In addition to the canonical molecules in the Wnt/β-catenin signalling pathway mentioned above, many other molecules also affect the signal transduction of the Wnt/β-catenin signalling pathway.

sFRPs, WIF, glypicans, Tiki, and Notum are well-known regulators of Wnt/β-catenin signalling that target Wnt proteins to affect signal transduction. sFRPs have a Netrin-related motif in the C-terminal region, WIF has a WIF domain in the C-terminal region, and both bind to Wnt proteins, thereby inhibiting Wnt signalling.^24,25^ Glypicans are located on the plasma membrane and bind to Wnt proteins to regulate their extracellular distribution and signal activity. Glypican-3 may have a CRD domain that interacts with the middle region of Wnt3a. Glypican-3 also promotes the coordinated activation of Wnt signalling when FZD is expressed at high levels. This effect is mainly caused by the interaction between GPC3 and FZD through the heparan sulfate (HS) chain.^26^ Both Tiki and Notum act as enzymes to inactivate Wnt proteins.^27,28^

In addition to targeting Wnts, some regulators can directly or indirectly activate FZD and LRP5/6 receptors, such as R-spondins, Norrin, and DKKs, and all of them are secreted proteins. The binding of LGR4/5 and R-spondin initiates the formation of a complex with RNF43/ZNRF3 and consequently inhibits the activity of Rnf43/Znrf3. Rnf43/Znrf3 are E3 ubiquitin ligases that are located on the plasma membrane and induce multiubiquitination of lysine. Rnf43/Znrf3 can ubiquitinate FZD, inducing FZD endocytosis and resulting in the downregulation of Wnt signalling.^29,30^ Norrin interacts with both the CRD domain of FZD4 and ectodomain ECD of LRP5/6 by simulating the finger-like loop of Wnt (the structure of Wnt interacts with the CRD domain of FZD), thereby activating the Wnt/β-catenin signalling pathway.^25^ DKK-1/3/4 prevent canonical Wnt signalling by binding to LRP5/6 or further forming a tertiary complex with the single transmembrane receptors Kremen 1 and 2, which induce the endocytosis of LRP5/6.^31^ However, DKKs are downstream targets of Wnt/β-catenin signalling, suggesting a negative feedback mechanism to govern WNT signalling.^32,33^

ROR and RYK are located on the plasma membrane, and both act as coreceptors of Wnt ligands in the Wnt/β-catenin signalling pathway. ROR-1 and ROR-2 have an extracellular CRD domain that closely resembles the CRD domain of the FZD receptor. RYK also has an extracellular domain that is homologous to Wnt inhibitory factor (WIF). Hence, ROR and RYK can compete with the Wnt ligand to inhibit activation of the Wnt/β-catenin signalling pathway.^34^

Spondin is a powerful Wnt agonist. The R-spondin protein family comprises four members with similar domains (Rspodin1-4).^35^ In 2004, Kazanskaya et al.^36^ found that R-spondin2 enhanced Wnt/β-catenin signalling in Xenopus embryos, and mouse Rspodin1-3 also had a similar effect. By inducing the receptor LGR4/5 to bind to Znrf3/Rnf43, R-Spondin, the negative feedback regulator of Wnt, removes the two E3 transmembrane ligases from the membrane, thus weakening the ubiquitination of Frizzled and significantly increasing the transmission of Wnt/β-catenin signals.^29,37^ Norrin protein can also bind to Frizzled class receptor 4 (FZD4) and LRP5/6 to form a ternary complex and promote the downstream signal transmission of Wnt/β-catenin.^38,39^

However, the Wnt/β-catenin signalling pathway is not only positively activated by the above factors but also inhibited by many negative feedback regulators, such as Notum, Tiki, DKKs, Wise/SOST, IGFBP-4, sFRPs, WIF1, and ZNRF3/RNF43. Some other molecules also regulate Wnt signalling by processing components in the pathway, such as PP2A and PP1. PP1 and PP2A bind to AXIN and APC, respectively, resulting in the dephosphorylation of AXIN and β-catenin. The dephosphorylation of β-catenin contributes to β-catenin accumulation in the cytoplasm, playing a positive role in Wnt signal transduction.^40^

In addition to the above regulatory factors, crosstalk between Wnt/β-catenin and other pathways also regulates canonical Wnt signalling in different ways (Table 4).

#### Noncoding RNAs that regulate the Wnt/β-catenin pathway

MicroRNAs are noncoding endogenous small RNAs. By pairing the 3′-untranslated regions (3′-UTRs) of target mRNAs, microRNAs participate in the regulation of Wnt/β-catenin signalling, and different miRNAs have different effects on the Wnt/β-catenin signalling pathway.^41,42^

Some miRNAs enhance the Wnt/β-catenin pathway by inhibiting the negative regulatory factors of the Wnt/β-catenin pathway. miR-100 and miR-125b synergistically inhibit five Wnt antagonists (DKK1, DKK3, ZNRF3, RNF43, and APC2).^43^ Similarly, miR-31 directly inhibits the negative regulatory factors of Wnt, namely, DKK1, GSK3β, and AXIN1, to stimulate canonical Wnt signalling.^44,45^ In oesophageal squamous cell carcinoma, miR-455-3p participates in the activation of canonical Wnt signalling by simultaneously targeting and inhibiting multiple negative regulatory factors (e.g., DKK3 and GSK3β) of the Wnt/β-catenin signalling pathway.^46^ miR-29 directly inhibits four factors in the Wnt/β-catenin pathway (GSK3β, CTNNBIP1, HBP1, and GLIS2).^47^ In CD133^+^ liver cancer stem cells (CSCs), miR-1246 inhibits AXIN2 and GSK3β, two important components of the β-catenin DC.^48^ MiR-128-3p simultaneously inhibits the expression of AXIN1, SFRP2, and WIF1 to enhance the activity of the Wnt/β-catenin signalling pathway, thus promoting non-small cell lung cancer (NSCLC) metastasis.^49^ MiR-374a promotes breast cancer metastasis by specifically inhibiting the expression of WIF1, PTEN, and WNT5A, which are negative regulators of Wnt.^50^ MiR-22-3p and miR-22-5p act together on negative regulatory factors (SFRP2 and PCDH15) of Wnt signalling, resulting in Wnt signalling pathway activation.^51^ APC is an important component of the degradation complex. However, miR-106a-3p, miR-494, and miR-155 directly target APC, promoting the accumulation of β-catenin in the nucleus and upregulating the active transcription of the target genes c-Myc and cyclin D1.^52–54^ Highly expressed miR-92a-3p reduces the ubiquitin-mediated degradation of β-catenin by directly inhibiting FBXW7 and MOAP1 and promoting the progression of colorectal cancer (CRC).^55^ MiR-182-5p directly inhibits the expression of FOXO3a, preventing its binding to β-catenin, enhancing the interaction between β-catenin and TCF4, and then promoting canonical Wnt signalling.^56^

Other miRNAs play a negative role in the regulation of the Wnt/β-catenin pathway. MiR-199a-5p acts on Wnt pathway signalling factors (FZD4, JAG1, Wnt2), upregulating the expression of AXIN2, MYCA, and CCND1 and inhibiting the Wnt/β-catenin pathway.^57^ MiR-384-5p suppresses the expression of FZD1, FZD2, Tgrbr1, and LRP6 and decreases the phosphorylation of GSK3β^ser9^ and β-catenin, preventing myocardial fibrosis induced by Wnt activation.^58^ MiR-4496 directly targets β-catenin to downregulate its expression.^59^ MiR-181a-5p inhibits the 3′-UTR activity of β-catenin and TCF4, thus reducing the expression of cyclin D1 and AXIN 1, target genes of the Wnt/β-catenin pathway, and plays a tumour suppressor role in CRC.^60^ In hepatoma cells, miR-125b-5p directly targets the downstream gene STAT3 to enhance β-catenin phosphorylation.^61^ In infertility and abortion, miR-200c negatively regulates FUT4 to inhibit α1-catenin on CD44, inactivating the downstream Wnt/β-catenin signalling pathway, and reducing the proliferation and adhesion of endometrial cells.^62^ MiR-708-5p upregulates the expression of the tumour suppressor CDH1 and inhibits the Wnt/β-catenin pathway via the interaction between CDH1 and β-catenin.^63^

LncRNAs also have a strong regulatory effect on Wnt/β-catenin, and most lncRNAs, such as FAST, JPX, LALR1, Linc00210, CCAL, NEAT1, and Lnc-β-Catm, directly or indirectly enhance the stability of β-catenin, thus activating the canonical Wnt pathway. In human embryonic stem cells (hESCs), the conserved lncRNA-FAST binds to the WD40 domain of the E3 ubiquitin ligase β-TrCP, inhibiting the ubiquitination of β-catenin by β-TrCP, resulting in the accumulation of β-catenin in the nucleus.^64^ In metastatic tissues of lung cancer, lncRNA-JPX inhibits the expression of GSK-3β and promotes the transfer of β-catenin from the cytoplasm to the nucleus.^65^ In addition, lncRNA-LALR1 in hepatocytes reduces the expression of AXIN1 by recruiting CTCF from the promoter of AXIN1. The decreased expression of AXIN1 suppresses β-catenin phosphorylation, enhancing the transcription of c-Myc and Cyclin D1.^66^ The lncRNAs Linc00210 and lncRNA-CCAL inhibit the activity of CTNNBIP1 and activator protein-2α (AP-2α), respectively, and block the binding of CTNNBIP1 and AP-2α to β-catenin, enhancing the interaction between β-catenin and the TCF/LEF complex.^67,68^ LncRNA-NEAT1 interacts with DDX5 and promotes the formation of a complex between DDX5 and β-catenin, enhancing gene transcription.^69^ Lnc-β-Catm is a highly transcribed lncRNA in hepatocellular carcinoma that combines with β-catenin and EZH2 to promote β-catenin methylation and further enhance β-catenin stability.^70^ In addition, some lncRNAs indirectly regulate the Wnt/β-catenin pathway by targeting microRNAs. The lncRNAs MIR100HG and MIR22HG are the host genes of miR-100/125b and miR-22-3p/5p, respectively, activating the Wnt pathway by upregulating the expression of these miRNAs.^43,51^ The lncRNA CRNDE enhances Wnt/β-catenin pathway signalling by inhibiting miR-181a-5p expression.^40^ In gastric cancer, the novel lncRNA LINC01133 targeting miR-106a-3p plays an indirect role in inhibiting the Wnt signalling pathway.^52^

#### Newly discovered molecules in the Wnt/β-catenin pathway

Recently, several potential regulators of the Wnt/β-catenin signalling pathway have been gradually identified, such as Twa1, FOXKs, ICAT, and Kdm2a/b.

Lu et al.^71^ found that Twa1 activates the Wnt/β-catenin signalling pathway by facilitating β-catenin nuclear retention in a zebrafish model. Twa1 (two hybrid-associated protein no. 1 with RanBPM, also known as Gid8 (glucose-induced degradation protein 8 homologue)) is mainly located in the cytoplasm. Twa1 is targeted by the AXIN complex for ubiquitination and degradation in the absence of Wnt signalling. When Wnt pathway activation occurs, Twa1 translocates to the nucleus, and its conserved CRA (CT11-RanBPM) domain is required for its interaction with β-catenin, which promotes β-catenin nuclear retention and increases Wnt/β-catenin downstream target gene expression.^71^

FOXKs promote Wnt/β-catenin signalling by translocating DVL into the nucleus. The forkhead box (FOX) transcription factors FOXK1 and FOXK2 are nuclear proteins that are crucial for the transport of DvL into the nucleus.^72^ The forkhead-associated (FHA) domain and hydrophobic motif (Leu-137-Phe-145-Phe-154) within its adjacent region on FOXK2 are both required for the interaction of FOXKs and DVL2, and the interaction is regulated by DVL phosphorylation. The association of FOXK with DVL can be enhanced by Wnt signalling and is consistent with the improved DVL nuclear translocation capability, which plays a positive role in regulating the Wnt/β-catenin signalling pathway.^72^

Inhibitor of β-catenin and TCF (ICAT) is an 81-amino-acid β-catenin-binding protein initially identified as an inhibitor of β-catenin signalling because its overexpression blocks the formation of the β-catenin-TCF complex. Recently, Ji et al. found that ICAT is a natural inhibitor of APC that plays a positive role in Wnt signal transduction by blocking APC-mediated degradation of β-catenin. On the one hand, when the WNT pathway is activated, ICAT blocks the interaction of β-catenin-APC and inhibits the degradation of β-catenin by competing with APC for the binding site of β-catenin. On the other hand, ICAT also inhibits β-catenin degradation by blocking the β-catenin-mediated APC-AXIN interaction. Considering how Wnt signalling inhibits the function of the β-catenin DC is not entirely clear, Ji et al. conjectured that Wnt might hinder the function of the Wnt-induced DC by inhibiting the direct interaction between APC and AXIN.^73^

Kdm2a/b regulate Wnt/β-catenin signalling by modulating the methylation/demethylation of nuclear β-catenin. Kdm2a/b are demethylation modification enzymes located in the nucleus. When Wnt is activated, nonphosphorylated β-catenin enters the nucleus and is methylated at lysine residues within the fourth and fifth Arm repeats. Subsequently, the modified β-catenin forms a complex with the TCF/LEF transcription factor to activate transcription. To stop signal transduction, Kdm2a/b competes with TCF/LEF for β-catenin binding and removes methyl markers from β-catenin, which is subsequently degraded via ubiquitination. Notably, the destruction of the β-catenin/TCF complex by Kdm2a/b inhibits Wnt signalling even without demethylation.^74^

## Function of the Wnt/β-catenin pathway in diseases

The Wnt/β-catenin pathway plays critical roles in embryonic development and adult tissue homeostasis. The dysregulation of Wnt/β-catenin signalling often leads to many serious diseases, including cancer and non-cancer diseases.

### Wnt/β-catenin pathway in non-cancer diseases

**Fig. 3:**
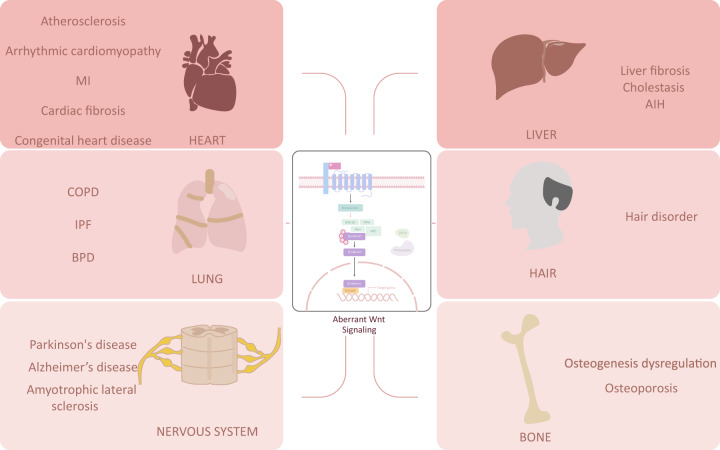
Wnt/β-catenin pathway and non-cancer diseases. Dysregulation of the Wnt/β-catenin pathway is involved in lung diseases, heart diseases, liver diseases, hair diseases, bone diseases, and nervous diseases. MI myocardial infarction, COPD chronic obstructive pulmonary disease, IPF idiopathic pulmonary fibrosis, BPD bronchopulmonary dysplasia, AIH autoimmune hepatitis

The Wnt/β-catenin pathway participates in the physical and pathological processes of the development of different organs and related diseases, including the development of the lung, angiocarcinoma, bone, neurons, and the liver and their corresponding diseases (Fig. 3).

#### Wnt/β-catenin pathway and lung physical and pathological processes

Common lung diseases, including chronic obstructive pulmonary disease (COPD), pulmonary inflammation, idiopathic pulmonary fibrosis (IPF), hyperoxia injury, bronchopulmonary dysplasia (BPD) silicosis, and lung cancer, are closely related to the Wnt/β-catenin pathway.

##### Wnt/β-catenin pathway in the physiological development of the lung

Canonical Wnt signalling plays an important role in the development and differentiation of the lung. It promotes the formation of the airway and alveoli.^75^ Two types of epithelial cells are found in alveoli: alveolar type 1 and type 2 cells (AT1 and AT2). AT1 cells mediate gas exchange, and AT2 cells secrete surfactants to maintain alveolar morphology.^76^ During airway differentiation, inhibition of the Wnt/β-catenin pathway contributes to the rapid directional differentiation of pluripotent stem cells into proximal airway epithelial cells, while activation of the Wnt/β-catenin pathway promotes formation of the distal airway epithelium.^77–80^ Alveoli are the main sites for gas exchange in the lungs, and the Wnt/β-catenin pathway plays a positive regulatory role in the development and differentiation of alveoli. LGT5^+^ mesenchymal cells located in alveoli can secrete Wnt3a to activate canonical Wnt signalling and promote alveolar differentiation of epithelial progenitor cells.^81^ Notably, Wnt5a, a common nonclassical Wnt pathway activator, after secretion by fibroblasts, activates the classic Wnt signalling pathway in AT2 cells and inhibits the transdifferentiation of AXIN2^+^ AT2 cells with stem cell activity into AT1 cells, thus maintaining the dryness of AT2 cells.^82,83^ When the epithelium is injured, activated Wnt/β-catenin signalling converts AXIN2^+^ myofibrogenic progenitor cells (AMPs) into mesenchymal alveolar niche cells (MANCs). MANCs and stable β-catenin promote the self-renewal of AT2 cells, facilitating alveolar regeneration.^77,84,85^ In PM2.5-induced lung injury, knockout of RAB6 activates the Wnt/β-catenin signalling pathway by inhibiting the secretion of DKK1, thus promoting the self-renewal of type II alveolar epithelial cells (AEC2) during lung repair.^86^ Folliculin (FLCN) deletion in pulmonary mesenchymal cells is the leading cause of cystic lesions in Birt-Hogg-Dubé (BhD) syndrome. A significant decrease in the activity of the Wnt/β-catenin signalling pathway was detected in lung tissues with FLCN knockout, indicating that inhibition of the Wnt signalling pathway is related to the abnormal development of alveoli.^87^

Together, the results show that the Wnt/β-catenin pathway promotes the differentiation of airway epithelial cells and AT2 cells, as well as the regeneration of alveoli.

##### Wnt/β-catenin pathway in lung-associated non-cancer diseases

Normal lung development and differentiation are inseparable from the regulation of the Wnt/β-catenin signalling pathway, but the imbalance of this pathway often promotes the occurrence of some lung diseases, including tumour and non-tumour diseases. This section focuses on the role of the Wnt/β-catenin pathway in non-tumour lung diseases.

COPD is a chronic disease characterized by chronic airway inflammation, airway remodelling, airflow obstruction, and loss of the alveolar parenchyma.^88^ Wnt/β-catenin signalling activity is reduced in the lung epithelial cells of COPD patients, and the mechanisms responsible for its downregulation include cigarette smoke and fibroblast-derived Wnt5a. (1) Cigarette smoke is the main risk factor for COPD. Cigarette smoke can reduce the expression of Frizzled receptor 4 (FZD4) in alveolar epithelial cells and enhance the phosphorylation of β-catenin, inhibiting epithelial proliferation and alveolar repair mediated by the classic Wnt signalling pathway.^5^ (2) The expression of fibroblast-derived Wnt5a is increased in COPD. However, after Wnt5a glycosylation, it negatively regulates Wnt/β-catenin signal transduction by reducing the stability of β-catenin, weakening wound healing and AT2 transdifferentiation.^89^ Notably, active Wnt/β-catenin signalling by the activator LiCl promotes the proliferation and survival of alveolar epithelial cells and improves alveolar repair, reducing the occurrence of emphysema, further confirming that Wnt/β-catenin reduces the occurrence of COPD.^90,91^ Recently, a Wnt-targeted RNA sequencing study of the COPD airway epithelium showed that the canonical Wnt signalling pathway is activated in COPD, which inhibits airway epithelial differentiation and barrier function and induces the upregulation of EMT-related genes.^92^

In contrast to COPD, the Wnt/β-catenin pathway is activated in the lung epithelium of IPF. IPF is a disease characterized by the progressive loss of lung function distinguished by severe pulmonary epithelial injury, fibroblast activation, extracellular matrix deposition, and distorted lung development.^93^ In IPF, abnormally activated Wnt signalling is detected, which manifests as follows: significantly increased expression of Wnt1, Wnt7b, Wnt10b, FZD2, FZD3, β-catenin, and LEF1 in the lung tissue, thereby promoting fibroblast proliferation and epithelial cell-mesenchymal transition, the pathological process of IPF.^94^ Abnormal activation of the Wnt/β-catenin pathway occurs in IPF fibroblasts, which stimulates the proliferation of mesenchymal cells and promotes the process of fibrosis.^95^ In addition, serum Kl6/mucin-1 (Muc1) overexpression in IPF mediates the activation of β-catenin and leads to the formation of the Muc1-cytoplasmic tail (cT)/β-catenin nuclear complex, which promotes the formation and proliferation of myofibroblasts.^96^

In addition, repeated lung injury inhibits the expression of chemokine receptor CXCR7 on lung capillary endothelial cells (PCECs) and recruits VEGFR1^+^ perivascular macrophages, causing excessive activation of the Wnt/β-catenin pathway in PCECs. Activated Wnt/β-catenin further promotes the expression of Jag1 in PCECs, stimulating Notch signalling in fibroblasts and enhancing fibrosis.^97^ Hyperoxia injury during lung development upregulates the expression of Wnt2b, Wnt5a, Wnt9a, and Wnt16, leading to excessive activation of Wnt/β-catenin in fibroblasts and AT2 cells, characterized by the accumulation of β-catenin and an increase in AXIN2 expression.^98^ Although active Wnt signalling is necessary for alveolar regeneration, it is strictly regulated by the body. Saccular stage hyperoxia injury not only decreased the expression of the alveolar formation genes FOXM1, MYB and MCM2 but also overactivated Wnt/β-catenin signalling. In particular, the highly upregulated Wnt5a in mesenchymal cells further mediates the occurrence of bronchopulmonary dysplasia (BPD) with impaired alveolar formation and septal thickening.^98–100^ In acute lung inflammation, the canonical Wnt signalling pathway in alveolar epithelial cells is usually inhibited by platelet-derived Dickkopf-1 (DKK1). Activated Wnt/β-catenin signalling inhibits the inflammatory response specifically as follows: ICAM-1/VCAM-1-mediated adhesion of neutrophils and alveolar epithelial cells is reduced, inhibiting the inflammatory response.^101^

#### Wnt/β-catenin pathway in cardiovascular diseases

Cardiovascular diseases, with high morbidity and mortality, are common and threaten human health. Wnt/β-catenin signal transduction is very active in the development and pathological remodelling of the cardiovascular system. The occurrence and development of many cardiovascular diseases are related to abnormal regulation of the Wnt/β-catenin signalling pathway.^30,102^

##### Wnt/β-catenin pathway in atherosclerosis

Atherosclerosis is characterized by the accumulation of lipid and fibre components on the arterial wall and leads to clogged or narrowed arteries. The CXCL12/CXCR4 axis can prevent atherosclerosis by regulating vascular permeability and the contractile phenotype in smooth muscle cells (SMCs). Activation of Akt/Wnt/β-catenin signal transduction by CXXL12/CXCR4 induces the expression of VE-cadherin and stabilizes its interaction with VE-cadherin-associated phosphatases to maintain the integrity of endothelial cells and favours a contractile phenotype over a synthetic phenotype in arterial SMCs, thus inhibiting atherosclerosis.^103^ However, several studies have shown that activated Wnt/β-catenin signalling also play a vital role in causing the deterioration of atherosclerosis and coronary heart disease by promoting the calcification of vascular SMCs and valve sclerosis.^104–106^ Arterial calcification is an important signal for cardiovascular disease, and inhibition of over-activated Wnt/β-catenin signalling may be a feasible strategy to modify vascular calcification and delay disease progression. Impaired lymphatic drainage of the arterial wall results in intimal lipid accumulation and atherosclerosis. R-spondins (RSPOs) are a family of four secretory proteins (RSPO1 to RSPO4). In atherosclerotic arteries, RSPO2 is enriched. Mechanistically, RSPO2 inhibits PI3K-AKT-eNOS signalling via LGR4.^107^

##### Wnt/β-catenin pathway in myocardial infarction

Myocardial infarction is defined as acute myocardial damage confirmed by abnormal cardiac biomarkers accompanied by acute myocardial ischaemia.

LRP5/6 deletion promotes cardiac ischaemia, while its downstream target, β-catenin, is beneficial in ischaemic injury. DKK-1 induces LRP5/6 endocytosis in cardiomyocytes, causing GPCR signalling disorder and promoting ischaemia-induced DNA damage in cardiomyocytes. IGFBP-4 binds to LRP5/6 in the presence of Wnt ligands to inhibit activation of the Wnt/β-catenin signalling pathway, which has a protective effect on myocardial ischaemic injury.^108^

##### Wnt/β-catenin pathway in arrhythmic cardiomyopathy

The pathogenesis of arrhythmic cardiomyopathy mainly includes haemodynamic changes, myocardial mechanical remodelling, and myocardial electrical remodelling.^109,110^

Ankyrin-B regulates arrhythmic cardiomyopathy through the Wnt/β-catenin signalling pathway.^110^ The localization of β-catenin in cardiomyocytes is related to the direct interaction with AnkB. AnkB-deficient animals have sinus bradycardia, a prolonged QT interval, and other myocardial electrical phenotype changes, promoting myocardial mechanical remodelling as well as premature death, which is related to the low expression of β-catenin in the intercalated disc and high expression in the cytoplasm caused by the loss of AnkB. GSK-3β inhibitors improve the cardiac function of AnkB-deficient ACM mouse models, and the use of these inhibitors may become a new prevention and treatment strategy for ACM.^111^

##### Wnt/β-catenin pathway in cardiac fibrosis

Cardiac fibrosis, which is common in various heart injuries, can significantly reduce tissue compliance and disrupt cardiac conduction. The absence of β-catenin in cardiac fibroblasts can reduce myocardial hypertrophy and post-TAC fibrosis and improve cardiac function.^112^ The Wnt/β-catenin signalling pathway is a key downstream molecular pathway of TGF-β-mediated myocardial fibrosis. TGF-β stimulates Wnt secretion and activates the Wnt/β-catenin signalling pathway through the TAK1 pathway to promote myofibroblast differentiation, which leads to myocardial fibrosis.^113,114^

##### Wnt/β-catenin pathway in other cardiovascular diseases

LRP5 regulates body fat distribution through the canonical Wnt signalling pathway and affects the occurrence of cardiometabolic disorders, which is consistent with the increased prevalence of cardiovascular disease in subjects with abdominal obesity.^115^

Aortic valve stenosis (AVS) is characterized by leaflet calcification and stiffening, eventually inducing left ventricular obstruction and ischaemic injury.^116^ In AVS patients, DVL2, GSK3β, β-catenin, and SFRP2 are upregulated in the stenotic aorta.^117,118^ Further investigations are encouraged to explore changes in downstream Wnt/β-catenin gene expression to eventually develop potentially propitious pharmacological drugs for AVS.

#### Wnt/β-catenin pathway in bone disease

Osteocytes play a central role in regulating bone metabolism and can regulate the balance of bone formation and bone resorption in bone modelling and remodelling through endocrine and autocrine methods.^119–122^ The canonical Wnt signalling pathway affects the proliferation and differentiation of mesenchymal stem cells (MSCs) and osteoblast progenitor cells, as well as the bone resorption of osteoclasts, contributing to bone formation and playing an indispensable role in the maintenance of bone homeostasis.^119^ In addition, other signalling pathways, such as the noncanonical Wnt, JAK/STAT, and Hedgehog (Hh) pathways, exhibit signalling crosstalk with the Wnt/β-catenin signalling pathway, jointly achieving the regulation of bone homeostasis. The Wnt signalling pathway has become a promising target for the development of new bone synthesis drugs.^123,124^

##### Novel regulators of bone formation related to the Wnt/β-catenin signalling pathway

**Regulation of bone formation and differentiation by Wnt players**: Doxycycline induces Wnt7b expression in an osteoblast cell line, and the high expression of Wnt7b in aged bone promotes the formation of trabecular and endosteal bone and increases the mineral density of the bone callus during fracture healing.^125^ Wnt1 in late osteoblasts and osteocytes regulates the function of osteoblasts and promotes bone homeostasis.^126^

Bioactive oxidized phospholipids (oxPLs) bind to LRP6 in bone marrow mesenchymal stem cells (BMSCs) and induce their endocytosis, contributing to the inhibition of Wnt signalling and diminishing osteoblast differentiation ability.^127^ CXXC5 inhibits bone formation and osteoblast differentiation via interactions with Dvl, which blocks the signal transduction of Wnt/β-catenin.^128^

In mouse femur tissues, the expression of miR-129-5p is negatively correlated with osteoblastic differentiation markers. MiR-129-5p binds to TCF4, inhibiting osteoblast differentiation.^129^ In human osteoblasts, miR-483-3p directly binds to DKK2, increases the expression of β-catenin and cyclin D1, and affects the bone formation process by impacting osteoblast proliferation, preosteoblast differentiation into mature osteoblasts, and new bone matrix formation.^130^ In BMSCs and preosteoblast MC3T3-E1 cells, miR-376b-3p directly targets YAP1 to suppress YAP1 expression, and circ_0024097 serves as a ceRNA to rescue YAP1 by absorbing miR-376b-3p, leading to activation of the Wnt/β-catenin signalling pathway and cell differentiation, which attenuates osteoporosis.^131^ In osteoblast cell lines, YAP stabilizes β-catenin and promotes nuclear β-catenin-mediated osteogenesis.

ZBP1 increases osteogenesis but suppresses the adipogenesis of MSCs, participating in a positive feedback process in bone differentiation regulation by the canonical Wnt signalling pathway. The absence of Wnt signalling inhibits the nuclear transport of β-catenin mediated by ZBP1, resulting in a decrease in Runx2 and Sp7 (osteogenic factors) expression in mouse bone marrow-derived MSCs.^132,133^

DHCR7 and INSIG1/2 are related to the effect of cholesterol metabolism on bone formation. Primary osteoblasts deficient in DHCR7 accelerate osteogenic differentiation by upregulating the expression of Col1a1 (osteogenic factor), while INSIG1/2-deficient cells have the opposite effect. β-Catenin binds to the Col1a1 promoter region, and this combination is related to the activity of Wnt/β-catenin signalling, indicating that the canonical Wnt signalling pathway is involved in the bone phenotype of DHCR7 and INSIG1/2 defects.^134^ R-spondin2 (RSPO2), an activator of the canonical Wnt signalling pathway, plays a vital role in regulating the production and mineralization of osteoblasts. We showed that RSPO2-deficient mice have reduced active β-catenin signalling and a decreased mineral deposition rate and osteoblast number, similar to the phenotype observed with the knockout of LRP5/6.^135^ Therefore, the canonical Wnt signalling pathway is considered the main mechanism of RSPO2 regulation, and it has been proposed that it may be a more precise bone mass regulator.^136^ Gα_s_ signalling simultaneously inhibits Hedgehog (Hh) signalling and enhances Wnt/β-catenin signalling. Bone formation depends on the maintenance of HH and Wnt/β-catenin signalling levels in a certain range.^137–139^

##### Wnt pathway and bone disease

Osteoporosis is the reduction in bone formation and increase in bone resorption. Osteoporosis has the characteristics of low bone mass, bone structure degradation, and easy fracture. Cortical bone determines bone strength; thus, cortical bone fragility is also a susceptibility factor for fractures.^140,141^ Inflammation plays an important role in tissue regeneration and bone loss. The current treatment for osteoporosis is mainly the inhibition of bone resorption.^142^ The regulation of some molecules in bone diseases can provide new ideas for the treatment of osteoporosis and other bone-related diseases.

**Positive bone mass regulators:** Local COX-2 overexpression promotes the differentiation of MSCs into osteoblast progenitor cells and inhibits their differentiation into chondrocyte progenitor cells. Studies have shown that local COX-2 overexpression increases the number of CD90^+^ mouse skeletal stem cells (mSSCs) at the fracture site and targets CD90 + mSSCs to enhance canonical Wnt signal transduction, which results in the promotion of intramembranous osteogenesis and inhibition of intrachondral osteogenesis.^143^

CD39 secreted by gingiva-derived mesenchymal stem cells (GMSCs) promotes bone formation through the Wnt/β-catenin signalling pathway. GMSCs are progenitor cells with immune regulation, repair, and regeneration capabilities. Previous studies have shown that GMSCs play an important role in the treatment of autoimmune diseases or inflammatory diseases, such as inhibiting the production of osteoclasts and bone erosion in autoimmune arthritis.^144^ A study by Wu et al. showed that the transcription level of Wnt3a in GMSCs was high, and the CD39-specific inhibitor POM-1 inhibited the expression of Wnt3a and reduced the level of β-catenin, ultimately leading to loss of the osteogenic ability of GMSCs. They also confirmed that transplanted GMSCs could be distributed in the bone marrow and might represent an effective method for the treatment of osteoporosis and other bone-related diseases.^145^

Highly sulfated glycosaminoglycans (sGAGs) regulate bone homeostasis by interfering with the formation of the sclerostin/LRP5/6 complex. Sclerostin (SOST) is a secreted protein produced by osteocytes that binds to LRP5/6 to inhibit Wnt signal transduction, thereby inhibiting bone formation. sGAGs combine with SOST to restore the signal transduction effect of Wnts. GAGs are used to coat biomaterials, making it possible for implants used for fracture treatment to better fuse with bone structures.^146^ Ann-Kristin Picke et al.^147^ found that sulfated hyaluronic acid (sHA3) also had a strong affinity for SOST and verified that the TriLA scaffold coated with collagen/sHA3 improved the regeneration of bone defects in type 2 diabetic rats.

NELL-1 activates Wnt/β-catenin signalling in osteoblast and osteoclast precursor cells. In bone marrow stromal cells (BMSCs), NELL-1 induces an increase in β-catenin, thereby promoting osteogenic differentiation and inducing OPG expression. In osteoclast precursor cells, activation of the canonical Wnt pathway also has an anti-osteoclast generation effect, inhibiting bone resorption. Therefore, NELL-1 may become a potential target for bone formation/anti-bone resorption combined treatment for osteoporosis.^148,149^

**Negative bone mass regulators:** Treatment of osteoblasts using JAK inhibitors downregulates the expression of STAT3 and SOC3 and promotes Wnt/β-catenin signal transduction, improving the functions of osteoblasts and reducing bone loss.^150^ STAT3 induces the binding of SOC and β-catenin to promote its degradation. Downregulation of STAT3 and SOC3 expression promotes the stability of β-catenin and signal transduction.^151,152^

PHD2 deletion in osteocytes in osteoporosis models increases bone formation and decreases bone resorption. In a hypoxic environment, the activity of PHD2 in osteocytes is reduced and HIF signalling is enhanced, improving the activity of the canonical Wnt signalling pathway through the HIF-SIRT1-SOST-β-catenin signalling axis, thereby promoting bone formation mediated by the activated Wnt/β-catenin signalling pathway and inhibiting osteoclast bone resorption mediated by RANKL and OGP.^150,153,154^

Cortical bone fragility is a common feature of osteoporosis. Kiper et al. found that the lack of sFRP4 is one of the causes of cortical bone thinning. Deletion of sFRP4 activates both canonical and noncanonical Wnt signalling pathways. Activation of noncanonical Wnt signalling activates BMP signalling pathways, reducing the formation of cortical bone.^140,155^

##### Other bone diseases related to the Wnt/β-catenin signalling pathway

Psoriasis is an inflammatory skin disease mediated by elevated IL-17A levels in patient serum. IL-17A effectively inhibits the function of osteoblasts and osteocytes in patients with psoriasis. Studies have shown that IL-17A downregulates the expression of downstream target genes in the Wnt/β-catenin signalling pathway, such as AXIN2 and CCND1, in osteoblasts and osteocytes, leading to bone loss by inhibiting the activity of osteoblasts and osteocytes.^156^

Myelodysplastic syndrome (MDS) includes a series of clonal haematopoietic stem cell (HSC) diseases, which may be caused by changes in the bone marrow microenvironment. Stoddart et al. (2019) found that inhibiting canonical Wnt signal transduction in bone marrow stromal cells prevented the development of MDS and proposed that activation of β-catenin signalling in the bone marrow of MDS patients may be a new treatment strategy that reduces β-catenin levels or inhibits Wnt/β-catenin signalling.^157^

Fluoride induces osteogenesis to cause bone fluorosis. β-Catenin mediates fluoride-induced aberrant osteoblast activity, and osteogenesis and may be a therapeutic target for skeletal fluorosis.^158^

#### Wnt/β-catenin pathway in neurodegenerative disease

Neurodegenerative disease includes a group of diseases characterized by the loss of cells and neurons of the brain and spinal cord. Parkinson’s disease (PD), Alzheimer’s disease (AD), and amyotrophic lateral sclerosis (ALS) are the most common neurodegenerative diseases.

Activation of the Wnt/β-catenin pathway has positive significance for the treatment of PD and AD. However, aberrant activation of the Wnt signalling pathway is related to the pathogenesis of ALS.

##### Wnt/β-catenin pathway in PD

Wnt/β-catenin pathway activity is related to abnormal morphology and neuronal mitochondrial dysfunction.^159^ Multiple Wnt/β-catenin signalling-related genes are hypermethylated in the brains of PD patients, including its receptor LRP5, the transcription factor TCF7L2, the inhibitors FRZB, SFRP1, and SFRP2, and multiple target genes. The protein expression of 4 Wnt and neurogenesis-related genes (FOXC1, NEURG2, SPRY1, and CTNNB1) in midbrain dopamine (DA) neurons is significantly reduced in rat models of PD.^160^

In another study, AXIN-2 shRNA lentivirus particles were stereotaxically injected into the substantia nigra pars compacta (SNpc) of parkinsonian rats. Activated Wnt/β-catenin signalling enhanced net DAergic neurogenesis in parkinsonian rats by regulating pro-neural genes (Nurr-1, Pitx-3, Ngn-2, and NeuroD1) and mitochondrial biogenesis.^161^

The development of mesodiencephalic dopaminergic (mdDA) neurons and mdDA progenitors, including the nigrostriatal subset that preferentially degenerates in PD, depends on Wnt/β-catenin signalling.^162^ Ghrelin, an endogenous ligand for growth hormone secretagogue receptor 1a, plays a fundamental role in regulating energy homeostasis in PD. Wnt/β-catenin signalling is relevant to DAergic neuron differentiation induced by ghrelin.^163^

##### Wnt/β-catenin pathway in AD

AD is mainly characterized by neuronal loss, the deposition of amyloid-beta plaques, and the formation of hyperphosphorylated tau protein in neurons, particularly the cytotoxic effect of amyloid beta-peptide (Abeta).^164^

The Wnt/β-catenin pathway intervenes in the pathological process of AD. The Wnt/β-catenin pathway is downregulated in the brains of AD patients. First, LRP6 is dysregulated in the AD brain.^165^ LRP6 is downregulated in the AD brain, and deficiency in LRP6-mediated Wnt/β-catenin signalling contributes to synaptic dysfunction and amyloid pathology in AD.^165^ Second, DKK1 upregulation suppresses Wnt/β-catenin signalling in the AD brain. DKK1 is induced by β-amyloid (Aβ) and shifts the balance from canonical Wnt signalling to noncanonical Wnt signalling. Activation of noncanonical Wnt signalling enhances Aβ production, while activation of canonical Wnt signalling suppresses Aβ production.^166^ Third, GSK3β is activated in the AD brain, and the hyperphosphorylation of tau protein in AD patients is mediated by GSK-3β. In addition, the Wnt/β-catenin pathway in the prefrontal cortex of AD patients is impaired.^167,168^ Activation of Wnt signalling rescues neurodegeneration and behavioural impairments induced by beta-amyloid fibrils. This phenomenon indicates that compounds that mimic the Wnt/β-catenin signalling cascade may be candidates for therapeutic intervention in patients with AD.^169,170^

##### Wnt/β-catenin pathway in ALS

ALS is a neurodegenerative disease characterized by the progressive loss of motor neurons. Wnt/β-catenin signalling is involved in the neurodegenerative process. Three key signalling molecules in the Wnt/β-catenin signalling pathway—Wnt3a, β-catenin, and Cyclin D1—are upregulated at the mRNA and protein levels in the adult spinal cord of SOD1 (G93A) ALS transgenic mice and are related to gliosis of the adult spinal cord in ALS transgenic mice.^171^

The levels of Wnt3, Wnt4, FZD 2, FZD 8, Wnt2b, Wnt5a, FZD3, LRP5, and sFRP3 are increased in the human spinal cord tissue of ALS patients. An increase in the number of FZD2^+^ astrocytes is observed in the borderline between the grey and white matter at the ventral horn in ALS samples. The Wnt family of proteins—more specifically, FZD2 and Wnt5a—may be involved in human ALS pathology.^172^

#### Wnt/β-catenin pathway in liver disease

The liver is a “metabolic factory” in humans. The Wnt/β-catenin pathway is associated with several common liver diseases, including cholestasis, liver fibrosis, fatty liver, and polycystic liver disease.

##### Wnt/β-catenin signalling pathway in the liver physiological process

A distinctive physical feature of the liver is that differentiated hepatocytes proliferate to regenerate the liver during acute liver injury.^173^ The Wnt/β-catenin pathway is an important regulator that controls liver growth, metabolic liver zonation, and liver regeneration in liver injury.^174,175^

Acute liver injury induced by carbon tetrachloride leads to activation of the Wnt/β-catenin signalling pathway, which manifests as an increase in the level of the Wnt target gene AXIN2. Lineage tracing of peri-injury AXIN2 hepatocytes has shown that, during recovery, AXIN2+ liver cells regenerate and repair damaged substances.^176^ This finding demonstrated that the Wnt/β-catenin pathway restores tissue integrity after acute liver injury. In addition, during liver zonation, knocking down LRP5 and LRP6 causes defective liver zonation in mice during liver regeneration. This result indicated that the cell membrane segment of the Wnt/β-catenin pathway regulates liver zonation.^177^ In a two-thirds partial hepatectomy (PHx) model, a widely studied liver regeneration model, β-catenin is rapidly enriched and is then transported to the nucleus, helping to induce cell proliferation and target gene expression.^178^ Importantly, stable regulation of Wnt/β-catenin activity is the key to maintaining liver regeneration. When Wnt/β-catenin continues to be abnormally activated, it often leads to liver cancer development.^175^

##### Wnt/β-catenin signalling pathway in non-tumour liver diseases

The cholestasis Wnt/β-catenin pathway decreases bile acid (BA) synthesis, preventing the development of cholestatic liver injury and fibrosis after bile duct ligation (BDL).^179,180^

Autoimmune hepatitis (AIH) is a chronic inflammatory liver disease that regularly recurs when immunosuppression is reduced. Aberrant activation of hepatic dendritic cells (HDCs) is observed in AIH, and the deficiency of canonical Wnt/β-catenin signalling in HDCs is the main cause of abnormal HDC function. Reactivation of the Wnt/β-catenin signalling pathway by enhancing binding to the Wnt ligand restores the immunomodulatory phenotype of HDC, reducing the severity of AIH. Targeting the Wnt/β-catenin pathway may become a new therapy for AIH.^181^

#### Wnt/β-catenin in hair disorders

Under normal conditions, the growth of mammalian hair is cyclical and is divided into the growth, anagen, and resting phases. The maintenance of hair circulation depends on the activity of hair follicle stem cells (HFSCs) in the bulge area of the hair follicle. The canonical Wnt signalling pathway plays an important role in regulating the proliferation and fate determination of HFSCs.^182,183^

An imbalance in Wnt/β-catenin signalling causes HFSCs to lose dryness or not be activated correctly, leading to hair disease. Deletion of the LGR4 gene in skin epithelial cells inhibits the activation of canonical Wnt signalling in hair follicles, manifesting as a significant reduction in the expression of β-catenin, c-Myc, AXIN2, and LEF-1, inhibiting the activation of HFSCs and delaying the growth phase.^184^ Hairless (HR) is a transcription inhibitor that is mainly expressed in the skin. It inhibits the expression of specific target genes by interacting with nuclear receptors, thereby regulating the development of hair follicles and the hair cycle. The 5′-UTR mutation of the human HR gene causes a rare autosomal dominant alopecia disease: Marie Unna hereditary hypotrichosis (MUHH), which is characterized by an abnormal hair density on the scalp, eyebrows, eyelashes, or body. In a study of Hairpoor (HRHp) heterozygous mice (an animal model of MUHH), HR overexpression in epithelial cells and keratinocytes was found to block the Wnt/β-catenin signalling pathway by upregulating DKK1 expression during the growth phase, promoting the degenerative progression of hair follicles.^185^

Wnt3a and β-catenin in hair stromal cells accelerate the hair follicle cycle and promote hair regeneration by activating the Wnt/β-catenin signalling pathway.^186–189^ In addition, the transcription factor Twist1 upregulates the expression of downstream target genes of TCF4, such as HGF, VEGF, and IGF-1, by forming complexes with TCF4 and β-catenin, promoting the proliferation of dermal papilla cells and inducing hair follicle regeneration.^190^

The Wnt/β-catenin signalling pathway plays an important role in regulating pigmentation during hair growth. For example, hepatocyte growth factor (HGF) secreted by dermal white adipose tissue activates the Wnt/β-catenin signalling pathway and enhances the pigmentation of hair follicles by upregulating the expression of AXIN2, LEF-1, Wnt10b, and Wnt6.^191^ In addition, O3,4,5-tri-caffeoylquinic acid enhances pigmentation by activating β-catenin in follicular melanocytes and upregulating the expression of MITF, a target gene, in caffeoylquinic acid.^192^

### Wnt/β-catenin pathway in cancer

In 1982, the first WNT gene was identified and cloned as an oncogene in mouse breast cancer.^152^ Dysregulation of Wnt/β-catenin signalling is often caused by mutations of various components in the pathway, particularly mutations or silencing of the Wnt tumour suppressor, which occurs frequently in various cancer types, such as adenomatous polyposis coli (APC) mutations,^193^ Axin1/2 mutations,^194,195^ GSK3β deletions,^196^ inactivation mutations of E3 ubiquitin ligase RNF43,^196^ and Znrf3.^197^ β-Catenin mutation is the most common alteration in cancers.^198^ The APC gene was first identified as a mutated gene in familial adenomatous polyposis coli (FAP). APC mutations are also found in 80% of colorectal adenomas and CRCs and are one of the earliest mutations in the progression of colon cancer.^199–201^ APC allele deletion activates Wnt/β-catenin signalling by inhibiting the formation of the degradation complex, which drives CRC development, endows cancer cells with self-renewal growth characteristics and is related to therapeutic drug resistance.^202–204^

AXIN is a scaffold protein of the degradation complex, and mutation of AXIN1/2 substantially affects Wnt signal transduction activity. In addition to the APC mutation, an AXIN2 mutation is also common in CRC.^205–208^ Patients with hereditary Axin2 mutations develop CRC.^209^ Generally, APC mutations and classic mutations of β-catenin are more common in CRC,^210,211^ gastric cancer (GC), and endometrial carcinoma,^212^ while AXIN mutations primarily occur in hepatocellular carcinoma (HCC)^213^ and medulloblastoma with dysfunction of the Wnt pathway.^195,214^ AXIN mutations are present in ~10% of hepatocellular carcinomas (HCCs).^194^ In addition, adenovirus-mediated gene transfer of wild-type AXIN1 induces HCC cell and CRC cell apoptosis, which is blocked by APC, CTNNB1, or AXIN1 mutations.^215^ The infiltration level of CD4+ and CD8+ T cells decreases in HCC tissues because of AXIN1/CTNNB1 mutations, resulting in immune escape.^216,217^ Several new potentially functionally relevant AXIN1 mutations have also been found in advanced prostate cancer.^195^ GSK-3β inhibits Wnt signalling by promoting β-catenin phosphorylation and then inducing the degradation of β-catenin mediated by E3 ligase. Studies have found that GSK3B deletion leaves HSCs in a precancerous state, and progression to acute myeloid leukaemia (AML) is facilitated after GSK3A deletion.^196^ Although the mutation frequency of E3 ubiquitin ligase is lower than that of other WNT inhibitors, mutations of RNF43 (which mediates ubiquitin degradation of Frizzled) are still detected in the adenoma tissues of patients with GC,^218^ early-onset metastatic CRC,^219^ and Lynch syndrome.^220^ RNF43 mutations are highly enriched in GC with microsatellite instability (MSI), promoting the development of GC cell resistance to DDR radiation and chemotherapy.^221^ Accordingly, BRAF, ARID1A, RNF43, and KM2B with higher mutation frequencies can be used to predict the prognosis and distinguish MSI from MSS tumours, and they provide guiding significance for the clinical effect of immunotherapy in MSI CRC.^222^ Simultaneously, RNF43 and KRAS mutations show a synergistic effect in CRC progression.^223^ Wnt signalling activated by RNF43 mutation promotes tumour growth and a high recurrence rate in CRC patients.^224^ Recent studies have further shown that some mutations in RNF43, such as p.G659fs, do not affect β-catenin signal transduction, while the C-terminal truncated mutant has a WNT signal transduction function similar to that of wild-type RNF43.^225^ Znrf3 inactivation mutations have been found in pancreatic cancer^226^ and adrenocortical carcinoma,^227^ but relatively few studies have examined the role of Znrf3 inactivation mutations in tumours.

Activation of the Wnt/β-catenin pathway includes not only classic mutations in suppressor molecules but also mutations in CTNNB1. In hepatocellular carcinoma, CTNNB1-activating mutations [28% (34/123) to 40% (18/45)] are the most significant genetic changes in the Wnt/β-catenin pathway.^228,229^ In medulloblastomas, CTNNB1 is a common mutation (15/125, 12%).^230^ In patients with childhood medulloblastoma, CTNNB1 gene mutations are only found in patients who are nuclear β-catenin positive, while the nuclear accumulation of β-catenin seems to be a sign of a good prognosis in medulloblastomas.^231^ Mutation of CTNNB1 exon 3 (87.0%; 47/54) may be the driver of low-grade and low-stage endometrioid endometrial carcinoma in young women.^212^ In elderly patients with CRC, the expression of nuclear CTNNB1 may be associated with higher mortality.^210^ Wnt/β-catenin signals are dysregulated in almost all stages of tumorigenesis, from malignant transformation to metastasis, spread, and drug resistance. This signalling may disrupt cancer immune surveillance, promoting immune evasion and resistance to immunotherapies, including immune checkpoint blockers.^232,233^ In addition to the inactivating mutation of Wnt/β-catenin pathway components, abnormal secretion of Wnt ligands in many tumours can also lead to overactivation of the Wnt/β-catenin pathway (DKK1, Wnt3a, and Wnt5a).^6^

Wnt/β-catenin signalling is essential to maintain the undifferentiated state of the stem cell population in the intestinal epithelium^234–238^

Abnormal activation of the Wnt/β-catenin pathway by mutations in adenomatous polyposis coli, AXIN2, or β-catenin (CTNNB1) is responsible for the initiation of almost all colon cancers.^6,239^ Recently, researchers have reported that the Wnt/β-catenin pathway collaborates with AHCTF1 (also known as ELYS) to act as a superenhancer-mediated gene to drive colon cancer.^240,241^ In a study of 955 patients with stage I, II, III, or IV colon and rectal cancer from 1980 through 2004, CTNNB1 was associated with improved CRC-specific survival and overall survival only in patients with a BMI ≥ 30.^242^

A more comprehensive experimental evaluation is needed for the functional assessment of different derived cancer suppressor mutations, improving auxiliary diagnosis and treatments.

## Application of the Wnt/β-catenin pathway in the clinic

Constitutive activation and inactivation of the Wnt/β-catenin pathway extensively participate in the physiopathological process of numerous diseases, indicating that this pathway has practical value in the clinic, particularly in the diagnosis, treatment, and prognosis of diseases.

### Wnt/β-catenin pathway in disease diagnosis

Because of the rapid development of molecular biology in recent years, the application of genetic testing for tumour diagnosis has gradually increased. Wnt/β-catenin signal transduction is an important regulator of embryonic development and adult homeostasis and is also closely related to the occurrence and development of many diseases. Related components of the canonical Wnt signalling pathway play potential roles in disease diagnosis.

#### Wnt/β-catenin pathway as a biomarker in the diagnosis of tumour-related diseases

**Wnt/β-catenin pathway in the cell-free DNA (cfDNA) test for CRC** The traditional diagnosis of CRC has many limitations,^243–245^ such as low sensitivity, poor specificity, and painful and traumatic procedures. However, cell-free DNA (cfDNA) analysis, which overcomes these limitations, can provide direct evidence of residual disease and can be used as a minimally invasive detection method for patients with CRC.^246^ Li et al. found that plasma cfDNA-based testing can capture specific genetic changes in CRC. Studies have shown that, in the cfDNA of patients with stage III or IV CRC, the copy number of key genes related to CRC (RTK, PI3K, and Wnt) is increased, and the copy number of cfDNA changes (CNVs) is positively correlated with tumour progression. The copy number of RSPO2 related to the canonical Wnt signalling pathway is increased. Therefore, the alteration of Wnt and its related gene copy number in cfDNA can be used to diagnose CRC. This diagnostic method is simpler and more readily accepted by patients and can be used for the treatment monitoring and clinical care of CRC patients.

**Wnt/β-catenin pathway in the DNA methylation of tumour-related diseases** Abnormal DNA methylation is an important cause of epigenetic gene silencing. In many human cancers, tumour suppressor gene methylation is widespread.^247^

Samaei et al. detected the methylation status of the gene promoters of 10 negative regulators of the Wnt/β-catenin signalling pathway in 125 groups of colon cancer and adjacent tissue samples. They found that the promoter methylation level of these genes was higher in cancer tissues than in adjacent tissues, and a correlation was found with different clinicopathological characteristics, such as age, sex, and other factors.^248^

Pietsch et al. proposed a diagnostic method for Wnt-driven medulloblastoma. Studies have shown that activation of the canonical Wnt signalling pathway is mainly evaluated by detecting the β-catenin immunophenotype, and methylation subgroups are consistent with IHC.^231,249,250^ The four DNA methylation subgroups of medulloblastoma in the study included Wnt, SHH, Group 3, and Group 4. All samples in the Wnt subgroup had mutations in the third exon of the CTNNB1 gene, which is the most reliable single marker to identify Wnt-driven medulloblastoma. Therefore, methylation analysis and CTNNB1 mutation analysis may be helpful for the reliable identification of Wnt-driven medulloblastoma.^251^ Genome-wide analysis can also be used to detect the methylation status and aberrant activation of Wnt/β-catenin signalling in primary plasma cell leukaemia.^252^

Promoter hypermethylation of Wnt inhibitors is related to activation of the Wnt pathway. In ALL-derived cell lines and bone marrow mononuclear cells from ALL patients, 7 Wnt antagonists (sFRP1, sFRP2, sFRP4, sFRP5, WIF1, DKK3, and HDPR1) showed abnormal promoter methylation. Clinically, in a group of 261 patients with newly diagnosed ALL, abnormal methylation of Wnt inhibitors was associated with decreased 10-year disease-free survival (25% versus 66%, respectively; *P* < 0.001) and overall survival (28% versus 61%, respectively; *P* = 0.001). The results indicate the role of abnormal Wnt signalling in ALL and establish a group of patients with a significantly worse prognosis (methylated group).^253^

#### Wnt/β-catenin pathway in the diagnosis of non-cancer diseases

##### Potential role of Wnt antagonists as diagnostic biomarkers in non-cancer diseases

DKK1 is an independent risk predictor for acute coronary syndrome (ACS).^254^ In a study evaluating ACS patients who had received dual antiplatelet therapy, the serum DKK1 levels were positively correlated with cardiovascular death after ACS. DKK1 improved NFκB pathway signal transduction, enhanced the inflammatory response at the injury site,^255^ and promoted ischaemic injury by downregulating LRP5/6.^108^ The increase in DKK1 in patients was related to the DKK1-mediated inflammation loop. All the above findings suggest that DKK1 can be used as a biomarker for further clinical decision making for ACS patients.^256^

The bone density of patients with AD was lower than that of the control group.^257^ Fourteen bone-related biomarkers in elderly people with memory problems were not clinically diagnosed with asymptomatic AD, and the expression of TRAIL and DKK1 was elevated in the brain.^257,258^ Wnt signalling has a neuroprotective effect, and DKK1, as an antagonist of the canonical Wnt signalling pathway, is negatively correlated with changes in cognitive function.^259^ Therefore, TRAIL and DKK1 most likely predict cognitive decline and may become potential biomarkers for the diagnosis of AD.^260^

Teriparatide (TPD) is a PTH analogue that can be used to treat osteoporosis.^261^ After using TPD to treat osteoporosis for more than 12 months, the serum DKK1 level increased, and the therapeutic effect of TPD decreased. This finding confirmed that chronic stimulation of PTH in the osteoblast cell line might reduce its anabolic effects through excessive secretion of DKK1. The detection of serum DKK1 levels plays an important role in the formulation of further treatment options for postmenopausal osteoporosis.^262^

##### Potential role of β-catenin as a diagnostic biomarker in non-tumour diseases

TGase-1 is a keratinocyte differentiation marker related to the differentiation of keratinocytes, and its expression increases in psoriatic skin.^263^ Nuclear β-catenin accumulation increases in keratinocytes on the epidermal base of patients with psoriasis, upregulating the activity of the transglutaminase 1 (TGase-1) promoter and leading to abnormal proliferation and differentiation of keratinocytes in the skin of psoriasis patients. TGase1 and β-catenin, with abnormally high expression in the skin, serve as potential biomarkers and targets for the diagnosis and treatment of psoriasis.^264^

##### Wnt signal transduction in the occurrence of pneumonia and obesity

A meta-analysis showed that the genes associated with childhood pneumonia include the top networks related to Wnt signal transduction. The diagnosis of pneumonia susceptibility factors is meaningful for the prediction, diagnosis, and treatment of lung-related diseases.^265^

The Wnt/β-catenin signalling pathway and renin-angiotensin system may participate in the remodelling of subcutaneous adipose tissue in the initial stage of human weight gain. After overeating, Wnt/β-catenin signal transduction in adipose tissue is inhibited, manifesting as increased expression of inhibitors of the canonical Wnt signalling pathway, such as SFRP2, FRZB/SFRP3, and DKK3, and increased phosphorylation and total GSK-3β levels. These factors lead to a decrease in the level of β-catenin and promote adipogenesis or adipose tissue remodelling. Therefore, the detection of related genes may become a potential target for the pathological diagnosis of adipose tissue in humans, including those with obesity.^266^

### The Wnt/β-catenin pathway and disease treatment

Abnormal activation of the Wnt/β-catenin signalling pathway promotes the progression of many human diseases, including cancer and non-cancer diseases. Hence, the canonical Wnt pathway has become a very attractive therapeutic target in recent years (Table 3).

**Table 3:** A nonexhaustive list of preclinical and clinical trials of Wnt/β-catenin signaling

Classified	Agent name	Functional effects in disease	Disease tested	Development stage	Reference
Inhibitor of DKK1	DKK1 antibody	Prevented vascular calcification; reduced circulating SOST levels; corrected renal osteodystrophy	Chronic kidney disease	Preclinical	^268^
Inhibitor of CBP/β-catenin	PrI-724	Promoted the inactivation of resting hepatic stellate cells in vitro; caused macrophages to migrate to the liver during the regression of fibrosis	Hepatitis C Virus-related Cirrhosis	Phase 1	^273^
Wnt small molecule inhibitor	Acyl hydrazones (M-110, OICR62321H7)	Chelated intracellular iron ions; prevented the proliferation of cancer cells	Colorectal cancer	Preclinical	^344^
Hypomethylating agents	Decitabine	Induced DNA demethylation of Wnt/β-catenin pathway; restored the sensitivity of ovarian cancer patients to carboplatin	Platinum-resistant ovarian cancer	Phase 2	^345^
A recombinant fusion protein	Ipafricept (OMP-54F28)	Blocked the canonical Wnt signal transduction and further inhibited tumour growth by competitively binding Wnt extracellular signal with FZD.	Advanced solid tumours	Phase 1	^346^
Small molecule tankyrase (TNKS1/2) inhibitors	JW67, JW74	Both compounds suppressed in vitro proliferation of colorectal cancer cell; JW74 reduced the proliferation of SW480 in vivo and adenoma formation in Apc^Min^ mice.	Colorectal cancer	Preclinical	^347^
Small molecule CK1αactivators	Pyrvinium	Suppressed in vitro proliferation of various colorectal cancer cell lines; reduced adenoma formation in Apc^Min^ mice.	Colorectal cancer	Preclinical	^348,349^
Small molecule inhibitors blocking β-catenin/CBP interaction	ICG-001	Supressed the growth of tumour cells in both vitro experiments and mouse xenograft models of colon cancer and pancreatic ductal adenocarcinoma	Colorectal cancer and Pancreatic ductal adenocarcinoma	Preclinical	^350,351^

#### Non-cancer diseases treatment

The canonical Wnt signalling pathway is a therapeutic target for many non-cancer diseases, usually by inhibiting Wnt signal and β-catenin activity.

**Agents targeting the β-catenin-destruction complex** chronic kidney disease (CKD)-mineral and bone disorder (CKD-MBD) begins early in the course of kidney disease and is clinically detectable in stage 2 CKD.^267^ DKK1 antibody are involved in the pathogenesis of CKD-MBD, neutralization of DKK1 in CKD-2 mice after renal injury stimulated bone formation rates, corrected the osteodystrophy, and prevented CKD-stimulated vascular calcification.^268^ A multi-centre osteoporosis study conducted in five European countries found that DKK1 positively associated with whole-body bone mineral density (WBMD) in subjects, regardless of age and sex.^269^ several clinical studies on osteoporosis showed that both zoledronic acid and denosumab reduced bone resorption through upregulation the endogenous Wnt-inhibitors Sclerostin (SOST) and DKK1.^270,271^ Meanwhile, Teriparatide (TPD) stimulate bone formation also via increasing SOST and DKK1 in postmenopausal osteoporosis treatment.^262^ In addition, in solid organ transplant recipients (SOTr), low DKK-1 and increased secreted frizzled related protein-3 levels causing dysregulation of Wnt signalling were closely associated with cytomegalovirus (CMV) infection.^272^

**Inhibitors targeting β-catenin/TCF transcription complex** PRI-724 is a first-in-class small molecule compound that inhibits the interaction between β-catenin and CBP, and it inactivates resting hepatic stellate cells in vitro and causes macrophages to migrate to the liver during liver fibrosis. A phase I trial showed that patients with Hepatitis C Virus-related Cirrhosis were well tolerated by intravenous infusion of 10 or 40 mg/m^2^/d of PrI-724, and the liver histology and Child-Pugh (CP) scores were improved in several patients.^273^ The agent C82 suppresses the activity of Wnt/β-catenin through blocking the interaction of the protein CBP with β-Catenin. Although the clinical trial showed C82 had few clinical effects on systemic sclerosis (SSc), microarray analysis C-82 treatment provide the possibility that C-82 with longer treatment to promote fat regeneration in SSc skin.^274^ Similarity, in another clinical trials of SSc, the researchers found that rituximab (RTX) relieved symptoms of patients with SSc by upregulation of Dkk-1.^275^

Further, a novel Wnt inhibitor, SM04690 were proved to effectively relieve the pain of severe symptomatic knee osteoarthritis (OA) and appeared safe and well tolerated through phase I/II clinical trial.^276,277^

In addition, numerous studies have shown that the therapeutic effect of drugs that are not direct activators or inhibitors of Wnt signalling pathway is also depend on the activity of Wnt signalling.^278–281^ All above indicate that targeting Wnt signalling is an attractive strategy for the treatment of multiple diseases.

#### Cancer treatments

**Fig. 4:**
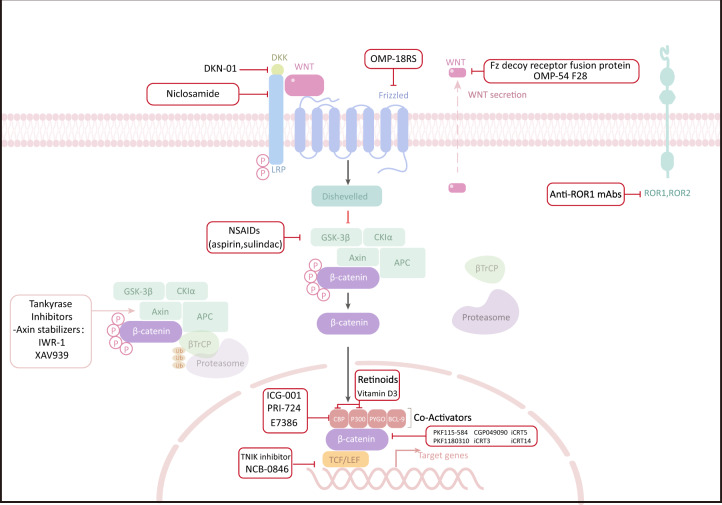
Canonical Wnt signalling pathway and pharmacological inhibitors under investigation in cancer

The dysregulation of Wnt/β-catenin signalling pathway is closely correlated with the development of cancers. Thus, medication targeting the pathway were developed in cancers. (Fig. 4) Figure 4 described a resumptive overview of Wnt/β-catenin signalling pathway targeted interventions in cancer therapy as follows:

CPG049090, PKF115-584 and PKF222-815 are natural compounds that have been identified to disrupt the interaction of β-catenin/TCF complex, suppressing the proliferation of colon cancer cells in vitro assays.^282,283^ In melanoma mice model, PKF115-584 restored immunocompetence which suppressed by β-catenin activation.^284^ TNIK is a key regulator of the β-catenin and TCF4 transcription complex, its pharmacological inhibition seems to be a promising therapeutic approach.^285^ In Apc min/+ mice, the TNIK inhibitor NCB-0846 effectively attenuated the frequency of intestinal tumorigenesis driven by Wnt signal. Also, NCB-0846 significantly reduced tumour formation by the same numbers of HCT116 and DLD-1 cells in an immunodeficient mouse model and PDXs (patient-derived xenografts). Importantly, the body weight of mice (immunodeficient xenograft model) decreased after treatment with NCB-0846, but gradually recovered.^286^ Besides, β-catenin responsive transcription (CRT) is another crucial therapeutic target in combating various cancers and CRT-inhibitory drugs (iCRT3, iCRT5, iCRT14) are specifically cytotoxic to human colon cancer cells and enhance the infiltration of T and NK cells in syngeneic mouse models of CRC by blocking β-catenin/TCF interaction.^287,288^ In the vitro experimental of head and neck cancer^289^ and triple-negative breast cancer (TNBC)^290^ also verified the antitumor effects of iCRTs. Moreover, in mantle cell lymphoma (MCL) which is resistant to genotoxic agents vincristine, iCRTs eliminated MCL-initiating cells very well.^291^ However, there are lacking enough vivo evidences to evaluate the antitumor potential of iCRTs. cAMP-responsive element binding (CREB)-binding protein (CBP) is a coactivator of Wnt/β-catenin-mediated transcription, and is another attractive target for cancer treatment. ICG-001, a preclinical agent, binding CBP and disrupting its interaction with β-catenin, is efficacious killing tumour cells in both vitro experiments and mouse xenograft models of colon cancer and pancreatic ductal adenocarcinoma (PDAC).^292,293^ PRI-724, an active enantiomer of ICG-001, has already entered Phase I clinical trials for treating colon cancer and advanced-stage pancreatic adenocarcinoma.^294^ Moreover, E7386 is the first-in-class orally active β-catenin-CBP antagonist that inhibits Wnt/β-catenin pathway in patient-derived tumour xenograft (PDX) model of HCC and now is in phase I clinical trials.(NCT03833700, NCT03833700).^295^

In all inhibitors of WNT signalling, niclosamide is FDA-approved drug and was used to treat taeniasis, showing inhibiting effect on tumours proliferation, stemness and metastasis by causing FZD1 receptor internalization and decreasing the protein level of DVL2, as well as targeting LRP6.^296–299^ Importantly, in these in vivo studies, niclosamide have shown limited toxicity providing hopeful optimism for Wnt inhibition in human cancer therapy.

In addition to directly affecting the function of tumour cells themselves, inhibitors of the Wnt signalling pathway also promote the activation and infiltration of immune cells in the tumour microenvironment and assist in improving the tumour-killing effect. Wnt/β-catenin signalling pathway has shown strong immunosuppressive function in a variety of tumours, and potentially become a novel target of tumour treatment.^300^ Using a mouse melanoma model, the researchers confirmed that the intrinsically active β-catenin signal of the tumour led to T cell rejection and resistance to anti-PD-L1/anti-CTLA-4 monoclonal antibody treatment.^301^ Simultaneously, the analysis of primary melanoma lesions with BRAF mutations showed that T cell infiltration was negatively correlated with β-catenin in tumour cells.^302^ Transmission of Wnt3a-β-catenin signal exhausted tumour infiltrating T cells, thus weakening their anti-tumour effects, and inhibiting the production of effector memory T cells.^303^

some Wnt inhibitors strengthen the Immune checkpoint blockade (ICB) therapy by combination therapy. A new specific Wnt inhibitor, a size-tuned nanocluster (CA_cluster_) improved the tumour response to the PD1/PD-L1 immune checkpoint blockade in melanoma and colon cancer.^300^ In the parental ID8 murine ovarian cancer model harbouring a knock-out of p53 (ID8p53) and MISIIR-Tag spontaneous ovarian cancer models, CGX-1321(Wnt inhibitor) increased infiltrating CD8^+^ T cells in the TME and decreased tumour burden.^304^

So far, some inhibitors of Wnt/β-catenin signalling pathway have become potential cancer treatment drugs and were in the stage of preclinical or clinical research. These drugs target different components of the canonical Wnt signalling pathway, which downregulates Wnt signalling transduction and thereby inhibiting cancer progression. Importantly, the use of Wnt activators versus inhibitors will be dependent on disease types, progression stage, and the nature of lesions.

## Discussion

Wnt/β-catenin signalling is an essential regulator in embryonic development and adult tissue homeostasis. Wnt/β-catenin signalling is initiated by extracellular WNTs and transduced by binding of the ligand to various receptors and coreceptors present at the cell membrane, activating intracellular transcriptional or nontranscriptional responses.

Numerous studies have shown that the abnormal regulation of Wnt/β-catenin signalling is involved in the occurrence and development of various diseases, such as cardiovascular disease, lung disease, liver disease, neurodegenerative disease, and tumours. Among these diseases, the potential common denominators in the pathologic mechanisms are processes such as cell proliferation and differentiation, inflammation, and fibrosis, and the role of Wnt/β-catenin signalling in these processes is well established, explaining why the Wnt/β-catenin signalling pathway plays a key regulatory role in these diseases.

In tumours, Wnt/β-catenin signalling was first found to be related to the ability to support cell proliferation and maintain stemness. Subsequently, several studies began to explore the function of Wnt/β-catenin signalling in angiogenesis, metastasis, chemotherapy resistance, and tumour metabolism. In recent years, with the emergence of immunotherapy, the indispensable role of WNTs in regulating T cell development and differentiation has been recognized.^305^ New studies have firmly established the importance of this pathway in regulating the expression of various checkpoints in immune cells and tumour cells to promote immune escape. In addition, dendritic cells and macrophages are regulated by Wnt ligands and Wnt signalling components, modulating innate immune and adaptive responses in various types of inflammatory diseases and tumours.^232,306^

The crucial role of Wnt/β-catenin signalling is not restricted to one type of tumour or cell. In addition to the effect on the biological behaviours of tumour cells, Wnt/β-catenin signalling is also sufficient to regulate the tumour microenvironment.^307^ Therefore, the development of different therapeutic strategies for the Wnt/β-catenin signalling pathway in cancers will greatly improve tumour treatment. Although modifying the activity of Wnt/β-catenin signalling is an attractive therapeutic approach, it remains challenging to develop activators or inhibitors of pharmacological agents regarding safety and selectivity.

Most of the studies presented in this review describe the role of interfering with or activating Wnt/β-catenin signalling in different disease models. Some results contradict other studies, making it difficult to draw firm conclusions about the potential therapeutic benefits of intervention. Sometimes a compensatory loop exists between different molecules because of the artificial manipulation of the key components or regulation of the pathway, which may not accurately reflect the real-world conditions. Furthermore, the Wnt/β-catenin pathway has many homologues at different levels and is involved in different signal transduction pathways, possibly leading to the redundancy and adaptation of organisms to a lack of specific genes. Therefore, researchers require elaborate clarification of the subsequent changed phenotypes after interfering with the Wnt/β-catenin pathway.

Because of the essential function of the Wnt/β-catenin pathway in human embryonic development and adult tissue homeostasis, the systemic administration of WNT or factors in the Wnt/β-catenin pathway may have extensive side effects. Thus, studies examining the development, safety and selectivity of targeted medicines are critical.

In recent years, studies have gradually focused on the ability of the Wnt/β-catenin pathway to assist cancer cells in evading the immune system; thus, modulators of the Wnt/β-catenin pathway have emerged to improve the efficacy of different immunotherapeutic agents in combination therapy, a finding that has already been researched in preclinical models. Furthermore, many small molecules have been reported to activate the Wnt/β-catenin pathway in vitro and in vivo. Some have shown promising therapeutic effects in preclinical models of disease. For therapeutic purposes, in studies of Wnt activators, topical treatment and target identification should be selected to address safety and selectivity problems.

Researchers have also identified novel regulators of the Wnt/β-catenin pathway, such as Twa1,^71^ TMEM59,^308^ GPR124, and Reck.^309^ How they affect and are affected by DC function and SCF-E3 ligase, as well as how they influence whole-cell proteins, including β-catenin, remain unknown. These thought-provoking questions will inspire studies that explore and discover new factors and functions of the classical pathway.

Regarding the recent developments concerning the Wnt/β-catenin pathway, the importance of the pathway in understanding different diseases is becoming the future direction for therapeutic research.

## Perspectives and challenges

Abnormal activation of Wnt/β-catenin signalling promotes the progression of various diseases and oncogenic transformation of tumours, making key factors involved in Wnt/β-catenin signal transduction attractive therapeutic targets.

Wnt/β-catenin targeted therapy is diverse. With the development of targeted drugs and combination therapy strategies, preclinical studies and clinical trials on the targeted intervention of Wnt/β-catenin signal transduction in malignant tumours have been gradually performed and are promising candidates for individualized therapy in cancer patients. Accordingly, drug-mediated regulation of Wnt/β-catenin signalling activity to induce liver regeneration would benefit this group of patients based on the key role of Wnt/β-catenin signalling in the pathobiology of non-tumour diseases such as steatohepatitis,^78,310^ cholestasis,^311^ and liver fibrosis.^158,159^ Tumour metastasis and chemotherapy resistance are the most important factors restricting the survival of cancer patients.

Tumours are insidious and often metastatic before they are detected. In CRC,^312^ ovarian cancer,^313^ and other tumours, activation of the Wnt signalling pathway is responsible for endowing and maintaining the ability of tumour cells to grow and metastasize.

Activation of the Wnt/β-catenin pathway can be used as a predictor of primary AA/P resistance in metastatic castration-resistant prostate cancer (mCRPC),^314^ and CRC is the main research object of Wnt inhibitors in clinical trials. Several inhibitors of the Wnt/β-catenin signalling pathway, such as ipafricept and OMP-131R10, have been shown to inhibit the growth and metastasis of advanced solid tumours in phase I or II clinical trials. Moreover, phase II clinical trials have demonstrated that decitabine increases cisplatin/carboplatin sensitivity in platinum-resistant ovarian cancer by affecting Wnt/β-catenin pathway.^307^ Therefore, the rational use of Wnt signalling pathway inhibitors is of great help to solve the clinical problems of tumour metastasis, recurrence and chemoresistance.

Because Wnt/ β-catenin signalling plays an important role in the aetiology of non-tumour diseases, small-molecule drugs targeting the Wnt/β-catenin signalling pathway have shown unique advantages in the treatment of these non-tumour diseases. DKK1 antibody effectively corrects renal osteodystrophy, while PRI-724, an inhibitor of CBP/β-catenin, inhibits the activation of hepatic astrocytes and delays the progression of liver cirrhosis. Wnt/β-catenin is also a potential therapeutic target for common but refractory diseases such as psoriasis, viral infections, and obesity.

A complete understanding of the specific role of Wnt/β-catenin in disease occurrence and progression can help guide the accurate use of these small-molecule drugs to maximize their efficacy and benefit more patients. Although Wnt/β-catenin signalling is an attractive target evaluated by clinical trials, there is a risk of off-target effects, such as the following: side effects such as diarrhoea, fatigue, nausea dysgeusia, and vomiting^315^ kidney injury; chronic kidney disease-associated complications^316^ and toxicities such as bone toxicity^315,317^ and intestinal toxicity.^318^ In addition, the appropriate timing of drug administration is critical, particularly in patient stratification and drug delivery.^305,319,320^

A more integrated experimental evaluation of mutations from different patient-derived tumours can be used to identify driver mutations from passenger mutations to guide treatment.
